# Cross-subject network investigation of the EEG microstructure: A sleep spindles study

**DOI:** 10.1016/j.jneumeth.2018.11.001

**Published:** 2019-01-15

**Authors:** Dimitris F. Sakellariou, Michalis Koutroumanidis, Mark P. Richardson, George K. Kostopoulos

**Affiliations:** a Division of Neuroscience, Department of Basic and Clinical Neuroscience, King’s College, London, UK; b Neurophysiology Unit, Department of Physiology, School of Medicine, University of Patras, Rio, Greece; c Department of Clinical Neurophysiology and Epilepsy, Guy's and St Thomas' NHS Foundation Trust, London, UK

**Keywords:** EEG networks, PCA, EEG microstructure, Sleep spindle networks, Graph theory, Pattern recognition

## Abstract

**Background:**

The microstructural EEG elements and their functional networks relate to many neurophysiological functions of the brain and can reveal abnormalities. Despite the blooming variety of methods for estimating connectivity in the EEG of a single subject, a common pitfall is seen in relevant studies; grand averaging is used for estimating the characteristic connectivity patterns of a group of subjects. This averaging may distort results and fail to account for the internal variability of connectivity results across the subjects of a group.

**New Method:**

In this study, we propose a novel methodology for the cross-subject network investigation of EEG graphoelements. We used dimensionality reduction techniques in order to reveal internal connectivity properties and to examine how consistent these are across a number of subjects. In addition, graph theoretical measures were utilized to prioritize regions according to their network attributes.

**Results:**

As proof of concept, we applied this method on fast sleep spindles across 10 healthy subjects. Neurophysiological findings revealed subnetworks of the spindle events across subjects, highlighting a predominance for occipito-parietal areas and their connectivity with frontal regions.

**Comparison with existing methods:**

This is a new approach for the examination of within-group connectivities in EEG research. The results accounted for more than 85% of the overall data variance and the detected subnetworks were found to be meaningful down-projections of the grand average of the group, suggesting sufficient performance for the proposed methodology.

**Conclusion:**

We conclude that the proposed methodology can serve as an observatory tool for the EEG connectivity patterns across subjects, providing a supplementary analysis of the existing topography techniques.

## Highlights

• Grand averages across subjects can distort connectivity results for a group.
• Within-group network variance may hold information for the EEG event under investigation.
• The proposed method can serve as an observatory tool, complementary to the existing topography EEG techniques.

## 1 Introduction

The microstructure of EEG has long been associated with neurophysiological functions and abnormalities of the brain. Microstructural graphoelements, such as the sleep spindle and K-complex (KC) appear to be linked with cognitive faculties (Fogel and Smith, 2011), intelligence (Knoblauch et al., 2003) and aging (Crowley et al., 2002). Moreover, certain EEG aspects of abnormal microstructural manifestations, such as the spike and wave discharge (SWD), play a key role in the identification of a variety of epilepsy-related syndromes (Pizzo et al., 2016) and disorders (Beniczky et al., 2013).

Computational analyses introduced in recent years, have aided the characterisation of EEG graphoelements. In this context, time-frequency techniques have shown the occurrence of fast sleep spindles to be prominent during non-REM stage 2 of sleep (NREM2) with temporal intra-cycle dynamics (i.e. with non-REM-REM transitions) and to progressively increase in number towards later cycles of sleep (Halász and Bódizs, 2013; Vyazovskiy et al., 2004). Through correlation techniques, spindles were shown to hold a contrariwise relation with delta waves (Dijk et al., 1993) and to associate both with slow and gamma-band oscillatory activity modulation (Ayoub et al., 2012; Spoormaker et al., 2011) with cross-frequency grouping (Steriade, 2006). Spindles have also been found to hold a dynamic relationship with other pathological and physiological EEG graphoelements, such as the SWD (Kostopoulos et al., 1981; Kostopoulos, 2000; Sitnikova, 2010) and the KC of NREM2 (Kokkinos and Kostopoulos, 2011; Kokkinos et al., 2013; Weigenand et al., 2016). Recent density and EEG-power topography studies of the latter showed a differentiation between patients with Alzheimer’s disease and older healthy individuals (De Gennaro et al., 2017).

Despite recent advancements, there has been a growing need for alternative approaches in the investigation of EEG graphoelements (Colrain, 2005; O’Reilly and Nielsen, 2014a, 2014b). Source localisation EEG (Dehghani et al., 2011) and intracranial electrocorticography studies (Andrillon et al., 2011; Piantoni et al., 2016) in healthy subjects and epilepsy patients respectively, dispute the generation of scalp spindles by widespread global and synchronous oscillations (Contreras et al., 1997; Loomis et al., 1937), and suggest them to be asynchronous activities of diverse focal sources in the cortex. Likewise, it is disputed whether the functional significance of the KC reflects an arousal phenomenon, a sleep-promoting response to an arousing stimulus or simply a marker of deep sleep that is hard to disturb (Colrain, 2005).

Regional characterization of EEG events is commonly studied via the distribution and correlation of the voltage and power of distinct regional EEG signals. Although techniques such as voltage topography, can evidently reveal crucial regional information (Kokkinos et al., 2010), they are also limited in their capacity to identify the origin of cortical activation. Specifically, the transmission of a cortical activation signal that is of low amplitude at onset, can occur through cortico-cortical circuits and potentially lead to maximisation at adjacent locations (Roland et al., 2014). In this case, voltage mapping gives a relative measure for the location of this onset activity. In the case of cortico-thalamo-cortical transmission however, these maxima are likely to occur at remote locations (Keller et al., 2014) suggesting that maximal amplitude at a given EEG location does not always indicate origin of onset. Further to this, such techniques are sensitive to EEG volume conduction and reference problems (Nunez et al., 1997a, 1997b).

We recently attempted to resolve such inconsistencies in a subject-specific technique that allows for the investigation of dynamic EEG interregional influences in the time and frequency domains (Sakellariou et al., 2016).

### 1.1 Aims

As an extension to the above method, we hereby propose a methodology for the cross-subject investigation of EEG connectivity. Commonly, EEG brain network studies derive from averaging the connectivities of subjects within groups. Here, we hypothesize the existence of internal meaningful structures that underlie their complex connectivity datasets and are likely to be hidden and not optimally represented in the grand average. By expressing the group data in a way that best explains the cross-subject variance, we aim to reduce its dimensionality i.e. the large number of interrelated variables, in order to reveal simplified structures that may underlie it (Joliffe and Morgan, 1992).

Specifically, for the EEG graphoelement under investigation, we aim to 1) reveal the key cross-subject network patterns and estimate how consistent these are across subjects, and 2) sort EEG regions according to importance, as in graph theoretic node analysis. The proposed methodology makes use of graph theory and pattern recognition techniques to provide a complementary view of the established EEG topography methods.

## 2 Methods

### 2.1 EEG network

As previously described in Sakellariou et al. (2016), an EEG network is defined here as the subset of discrete EEG-areas that appear to interrelate, due to the occurrence of an EEG graphoelement, such as the fast sleep spindle. A pair of EEG-areas is assumed to be functionally connected when the electrical activities of the two

• Exhibit significant phase consistency (Nunez et al., 1997a, 1997b)
• Are causal with each other i.e. Δφ≠0 (Nolte et al., 2004)

The connectivity between those areas can be either direct (cortico-cortical) or indirect (thalamo-cortical) however, distinguishing between the two cannot be determined from surface EEG data alone.

More specifically, for each subject the interregional dynamic connectivity levels between two areas were calculated using the imaginary part of coherency (Nolte et al., 2004) (eq. 1):

(1) $${Coh}_{ij}\left(f\right)=\frac{S_{ij}(f)}{{\left(S_{ii}\left(f\right)S_{jj}\left(f\right)\right)}^{\frac{1}{2}}}$$

where $s_{ij}(f)\equiv \langle a_{i}\left(f\right)a_{j}^{*}\left(f\right)\rangle$ the cross-spectrum of the (complex) Fourier transforms $a_{i}(f)$ and $a_{j}\left(f\right)$ of the time series ${\hat{a}}_{j}\left(t\right)$ and ${\hat{a}}_{j}\left(t\right)$ of channels i and *j*, respectively. The methodology we followed is amplitude-invariant while it allows for the association of connectivity values with the EEG graphoelements under investigation by presenting the former over the time and frequency domains. Finally, the method makes use of bootstrap resampling to infer statistical significance and is further described in a previous study (Sakellariou et al., 2016).

Here, a static network for each subject was calculated to reflect connectivities within a time-frequency window, in order to allow cross-subject estimations; In order to reflect connectivities that primarily concern the graphoelement of interest, static network values were calculated as the absolute average of the dynamic connectivity values over a time-frequency window for which the EEG graphoelement takes maximal power values for each subject. Specifically, the ranges of this time-frequency window were determined according to the event-related peak time and frequency as observed in the time-frequency analysis of each subject (Table 1 - Suppl Fig. 1). We previously showed in simulations (Sakellariou et al., 2016) that coherency estimates increase for frequencies that specifically relate with the characteristic frequencies of the stochastic processes under investigation. Therefore, it is reasonable to assume that meaningful connectivity activations will primarily occur at the same frequency window as the EEG graphoelement under investigation is neurophysiologically characterised by.

**Table 1:** Fast sleep spindles peak frequency characteristics.

Subjects	Number of spindle trials	Peak frequency (Hz)	Lower frequency limit (Hz)	Upper frequency limit (Hz)
01	120	15.20	14.00	16.45
02	279	13.45	12.30	14.65
03	150	14.05	12.80	15.30
04	109	13.15	12.05	14.30
05	240	14.55	13.45	15.70
06	317	13.45	12.40	14.55
07	141	13.70	12.65	14.70
08	93	13.55	12.60	14.60
09	122	13.10	12.15	14.00
10	76	13.75	12.65	14.85
Mean ± SEM	165 ± 26	13.80 ± 0.21	12.71 ± 0.19	14.9 ± 0.23

**Fig. 1:**
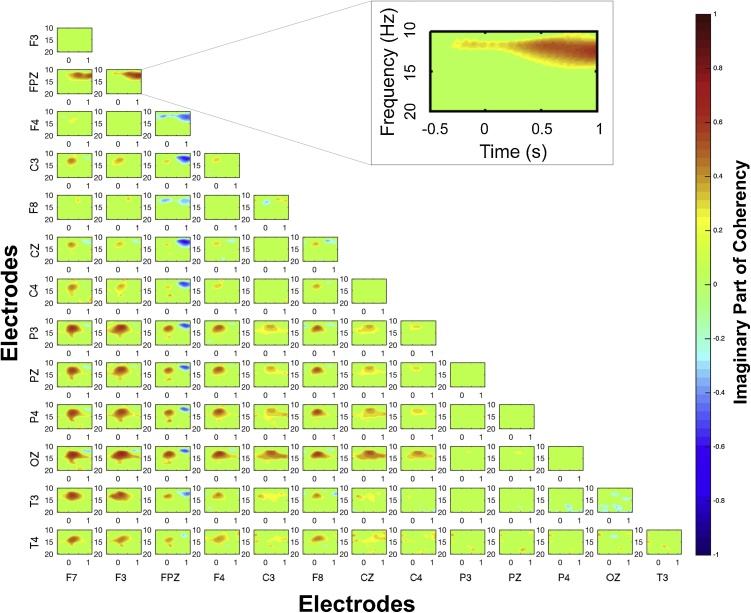
| Connectivity map. The event-related connectivity map of subject 7, as calculated according to Sakellariou et al., 2016. The detailed maps show significant (a = 0.05) connectivity values across all possible pairs of electrodes, across frequency and time. Each box represents EEG-connectivity between two different electrodes, as those are seen in the X–Y axes of the multigraph. X and y axes of each box represent time and frequency, respectively. Strength of interaction are shown in relation to colour intensity with different shades of red or blue (values shown in colorbar). Positive and negative information flow according to each box’s label is represented in warm (red) and cold (blue), respectively. Taking as example the box labelled as "F7-T4″, red colour refers to high level of interaction between the two regions with the information flowing from the F7 towards T4 brain region, whereas blue colour would stand for high level of interaction with the information flowing from the T4 towards F7.

### 2.2 Cross-subject network

The cross-subject network is defined here as the average of all normalised subject-specific EEG networks. Normalisation occurs across the range of [0,1] per standard feature scaling of the numeric variables. The cross-subject network provides a classical framework for the common aspects of the graphoelement network across subjects.

### 2.3 Network modules

As network module, we define here a pattern of interactions between a subgroup of EEG-areas. To further investigate potentially meaningful patterns i.e. patterns that best explain a large amount of the overall data variance in the cross-subject complex connectivity data, we used principal components analysis (PCA) techniques as seen in pattern recognition (Pearson, 1901). Specifically, PCA is applied to $G\in R^{n\times m}$, where *n* rows correspond to subjects and *m* columns to the connectivity values of each possible pair of electrodes after subject-specific mean normalisation calculated according to the methodology described above. PCA is mathematically defined as the orthogonal linear transformation of the original, possibly correlated variables into a set of linearly uncorrelated variables i.e. principal components (PC) (Joliffe and Morgan, 1992).

Generally, PCA is estimated by eigen-decomposition of the data covariance matrix (eq. 2) that is their tendency to vary together.

(2) $$cov\left(X,Y\right)=\sum_{i=1}^{N}\frac{\left(x_{i}-\bar{x})(y_{i}-\bar{y}\right)}{N}$$

where

$\bar{x}=mean(X)$ and $\bar{y}=mean(Y)$ random variables, and *N* is the dimension of the datasetAi,j=coν(i,j).The full Principal Components (PCs) decomposition (Eq. (3)) of a data matrix *G* can therefore be given as

(3) PC=GM

where *M* is a *p*-by-*p* matrix whose columns are the eigenvectors of *G^T^G, as G^T^G* itself can be recognised as proportional to the empirical sample covariance matrix of the dataset *G*. PCA finds the *k*-dimensional subspace of maximal variance in the data and PCs are extracted to represent patterns of the underlying structures of the variables, in this case the connections between EEG areas.

The number of principal components to be taken into account is usually determined in relation to the captured variance of the data. Simplistically, stable components are considered the ones with eigenvalues larger than 1 (the average eigenvalue will be 1, so PCs with eigenvalues larger than 1 are higher than average), while another non-criterion based approach is to consider the principal components that collectively explain over 80% of the total variance. Moreover, the quality of each component can be verified through the symmetry of distribution of the component scores around the mean. Here, we tested principal components for all of the above criteria.

A key weakness of standard PCA is that principal components are linear combinations of all original features and therefore most of the eigenvectors’ coefficients are non-zero. This makes the interpretation of PCs difficult when they are still constructed by all variables (Cadima and Jolliffe, 1995).

#### 2.3.1 Sparse PCA

Sparse PCA (SPCA) is a technique that aims to resolve these interpretation difficulties of PCA due to the large number of explicitly used variables. SPCA extends standard PCA and produces modified principal components with sparse loadings by finding linear combinations that contain just a few input variables (Zou et al., 2006). Here we make use of an Inverse Power Method for the calculation of SPCA (IPM-SPCA) described by Hein and Buhler (Hein and Bühler, 2010). IPM-SPCA considers SPCA a non-linear eigenproblem and allows for selection of the number of non-zero features while still explaining most of the data variance. Moreover, without any sparsity constraint, SPCA should reduce to PCA. Therefore, it is reasonable to select the number of non-zero loadings in order to (1) be lesser than the ones in standard PCA (2) capture the desired percentage of the overall data variance with lesser components.

The pipeline of the analysis is presented graphically in Supplementary Fig. 2.

**Fig. 2:**
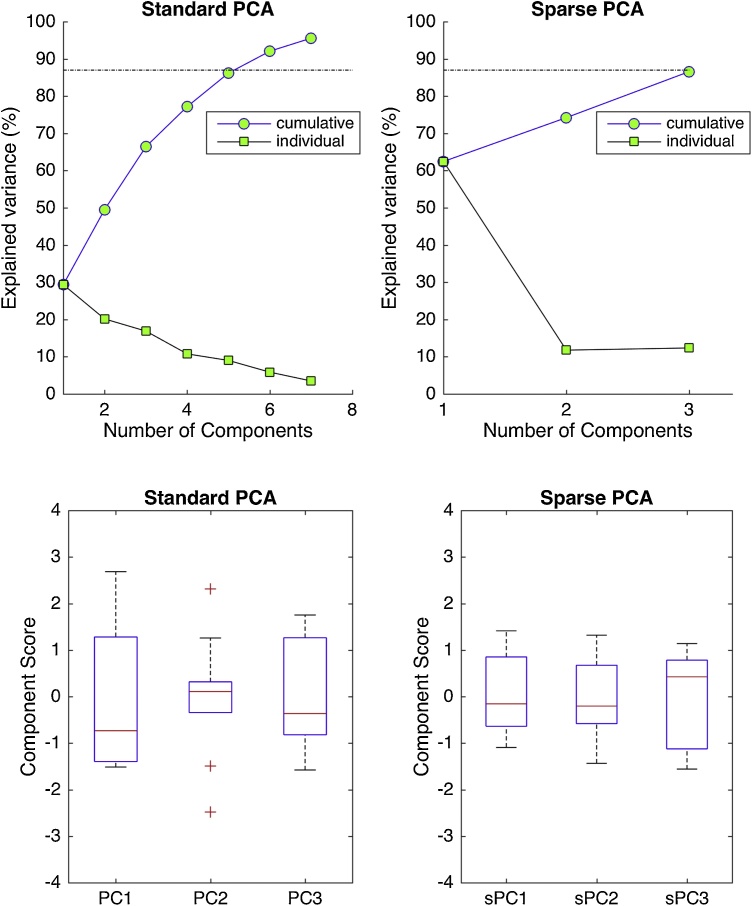
PCA and IPM-SPCA Scree and Box Plots (Top) Proportions of cumulative and individual variance explained by the principal components of PCA and IPM-SPCA (top). The first three sPCs of IPM-SPCA account for more than 85% of the variance. Standard PCA would require more than 5 PCs to express the amount of variance that the three sPCs account for. (Bottom) Representation of component scores via quartiles, with the bottom and the top of each box indicating the 25th and 75th percentiles, respectively. On each box, the central red mark indicates the median. Three outliers are observed for PC2. IPM-SPCA components showed no outliers and symmetrical distributions around zero suggesting satisfactory performance for the representation of the network properties across subjects.

### 2.4 Node influence metrics

#### 2.4.1 Global patterns of networks

Here, we consider the EEG sensors as nodes of the EEG network. To quantify the importance of each node in the cross-subject network, we calculated their involvement according to the following measures:

• Node strength (${NS}_{i}$) or weight (Eq. (4)) of a node *j*, that is the sum of connectivity values *w* of a node *i* with every other *j* node, against the number of all nodes *N*

(4) $${NS}_{i}=\frac{1}{N}\sum_{j\in N}w_{ij}$$

where *wij* = connectivity value for the i-by-*j* pair of nodes/electrode areas, *N* = total number of nodes.

• Node degree $d_{i}$ (Eq. (5)), that is the number of connections a node *i* sustains in the network,

(5) $$d_{i}=\sum_{j\in N}a_{ij}$$

where $a_{ij}$(eq. 6) is the status of the link i.e., connection *(i,j)* between the nodes *i* and *j* where $\left(i,j\in N\right)$ and

(6) aij=1, when link i,j exists 0, when link i,jdoes not exist

#### 2.4.2 Segregation patterns and position of nodes in the network

It is widely considered that central nodes have major impact in the functionality and the evolution of network dynamics. However, there is no unanimity on exactly what centrality is or on its conceptual foundations, and therefore there is little agreement on the proper procedure for its measurement (Freeman, 1977). Here we employ three centrality measures to estimate the segregation and position of a node in the network:

• **Betweenness centrality** (Freeman, 1977; Wang et al., 2008) depicts the role of a node as an intermediary connector based on the idea that nodes for which more information flows, will be higher in values of centrality. It essentially measures the importance of a node as a connector of other nodes, independently of the number of connections or otherwise degrees. As shortest path length, we define here the average number of steps along the shortest paths for all possible pairs of network nodes (Dijkstra, 1959). Since there can be several shortest paths between two graph nodes *i* and *j*, the centrality of node *u* is:

(8) $${c(u)}_{betweenness}=\;\sum_{i,j\neq u}\frac{n_{ij}(u)}{N_{ij}},$$

where $n_{ij}\left(u\right)$ is the number of shortest paths from *i* to *j* that pass through node *u*, and $N_{ij}$ is the total number of shortest paths from *i* to *j*. In our case where the network is undirected, the paths from *i* to *j* and from *j* to *i* count only as one path (divide Eq. (8) by two). To calculate the weighted betweenness we allowed edge weights to specify the length of the edges and determine the shortest paths between nodes *i* and *j*.

• **Closeness centrality** (Sabidussi, 1996) of a node is a measure similar to betweenness and it estimates the relative distance to other nodes or otherwise the ease of reaching out to other nodes. Closeness can generally be considered a measure of how fast information spreads from a node *u* to all other nodes sequentially. Closeness scales directly with the weight of connections and is calculated as the inverse sum of the distance from a node to all other nodes in the network,

(7) $${c(i)}_{closeness}={\left(\frac{A_{i}}{N-1}\right)}^{2}\frac{1}{C_{i}},$$

where $A_{i}$ is the number of reachable nodes from node *i* (excluding itself), *N* is the number of nodes in the network, and $C_{i}$ is the sum of weights from node *i* to all reachable nodes. Closeness is generally restricted to nodes within the largest component of a network. As in betweenness, to calculate the weighted closeness here we allowed edge weights to specify the length of the edges (strong and weak connections relating to short and long distances, respectively).

• **Eigenvector centrality** is a measure of prominence of importance in the network and it is proportional to the sum of neighbours’ centralities. Under this notion, it is more significant for a node to have important neighbours rather than having numerous unimportant neighbours. The eigenvector centrality of a node *i* is generally calculated as:

(9) $${c(i)}_{eigenvector}=\frac{1}{\lambda_{max}}\sum_{j\in N(i)}c_{j}=\frac{1}{\lambda_{max}}\sum_{j=1}^{n}A_{ij}c_{j}\;,\;i=1,2,\ldots ,n$$

where $A_{ij}$ the weighted adjacency matrix and $\lambda_{max}$ its the largest eigenvalue. The above directly lead to an eigenvector problem,

(10) $$Ac=\;\lambda_{max}c$$

where the eigenvector corresponding to the largest eigenvalue will be non-negative according the Perron-Frobenius theorem (Meyer, 2000) and is the one to reflect the eigenvector centrality scores. The scores are normalized such that the sum of all centrality scores is 1. Here, a weighted adjacency matrix that originates edge weights of the network is being used to calculate the weighted eigenvector centrality.

It is important to note that the above measures capture different ideas and aspects of a position in the network.

### 2.5 Subjects and analysis

Ten individuals (6 females) aged between 22 and 33 years (mean age 25.4 ± 2.6) volunteered to participate in this study. None of the participants reported any problem with their sleep (i.e. problems with sleep onset, apnea, insomnia or interrupted sleep patterns). None of the subjects were taking medication and none had any medical problems or reported history of neurological/psychiatric disorders. A seven day sleep report by each subject was requested to track possible implications in sleep patterns. Abstention from alcohol and caffeine was instructed for seven days up to the EEG recording day.

All subjects arrived at the laboratory one hour earlier than their usual bedtime according to the seven day sleep report and slept for one night in the sleep room, which is a soundproof and temperature controlled dark Faraday-cage room.

Sleep EEG recordings began after the subjects voluntarily turned off the sleeping room lights and woke up undisturbed in the morning. EEG recordings were monitored from an adjacent control room where the subjects could communicate verbally via installed microphones.

All subjects reported normal and undisturbed sleep the following day.

All procedures described were approved by the University of Patras Committee for Ethics in Research. All experiments were performed in accordance with relevant guidelines and regulations. Written and informed consent that outlined the procedures and purpose of the study was obtained from all subjects. All data created during this research are available and can be provided by the corresponding author upon request in line with participant consent agreements.

#### 2.5.1 EEG recording

All-night sleep EEG signals were acquired by the use of 58-tin electrodes according to the extended standard international 10–20 system (FP1, FPZ, FP2, F3A, F4A, F7, F5, F3, F1, FZ, F2, F4, F6, F8, C5A, C3A, C1A, CZA, C2A, C4A, C6A, T3, C5, C3, C1, CZ, C2, C4, C6, T4, T3L, TCP1, C3P, C1P, PZA, C2P, C4P, TCP2, T4L, T5, P5, P3, P1, PZ, P2, P4, P6, T6, CB1, P3P, P1P, PZP, P2P, P4P, CB2, O1, OZ, O2). For this study, an electrocap (ElectroCap International, Eaton, OH, USA) with 4.5 cm inter-electrode spacing was used. Earlobe and AFZ electrodes were used for reference and ground of the EEG channels, respectively. A bipolar derivation of oblique electrooculogram (EOG) was used to detect eye movements, for which electrodes were placed 1 cm above the right outer cantus and 1 cm below the left outer cantus, and muscle tone was tracked by placement of bipolar Electromyography (EMG) to the upper masseter muscle. Electrode impedances were kept at below 10 kOhms for most of the recording period. All electrophysiological parameters were originally AC recorded and amplified by a total gain of 1000. A band-pass 0.05–500 Hz filter was used while all signals were digitised via an AD converter with 16-bit resolution that resulted in 0.084 u V/LSB of accuracy. The sampling frequency for all recordings was set to 2500 Hz (Synamps system, Neuroscan, Charlotte, NC, USA), and all data were stored on hard disk drives. No use of notch filters was made. Subject movements were detected throughout the recording by a motion detector and all movement-events were stored into an event channel alongside the rest of the EEG recordings. For the connectivity analysis, we only used the 10–20 EEG montage electrodes.

#### 2.5.2 Sleep staging

Manual sleep staging was performed by a sleep expert by visual inspection of the EEG recordings along with EOG and EMG channels using the criteria of propositions of the AASM Visual Scoring Task Force and the DGSM Task Force (Berry et al., 2012; Rodenbeck et al., 2006), and keeping a display time resolution of one second (Table 2). The sleep latency was calculated from the analysis start to the first 30-s period of consecutive sleep events. The latency to sleep stage N1 (NREM1) was measured until the occurrence of 30.0 s of consecutive NREM1. The minimum REM duration was 240.0 s and the minimum interval between REM's is 60.0 s to be considered a REM period. The minimum slow wave sleep (SWS) duration is 240.0 s and the minimum interval between SWS's is 60.0 s to be considered a SWS period.

**Table 2:** Sleep staging analysis.

Subject	Total sleep time (TST) (min)	Sleep efficacy (%)	Sleep latency (min)	REM (%) of TST	N1 (%) of TST	N2 (%) of TST	N3 (%) of TST
01	411.5	91.5	9.5	19.4	12.4	47.8	20.5
02	413.5	80.5	3.0	15.0	3.6	52.6	28.8
03	287.5	78.4	9.5	12.2	19.5	40.2	28.2
04	580.5	92.1	6.0	18.3	10.2	54.2	17.4
05	384.0	96.5	2.5	16.3	1.8	51.2	30.7
06	484.0	93.8	4.0	23.8	3.5	56.7	16.0
07	561.0	87.0	4.0	16.3	11.1	58.7	13.8
08	340.0	85.4	8.5	17.5	3.8	51.9	26.8
09	527.5	97.5	2.0	25.2	4.0	48.8	21.0
10	538.0	91.8	1.5	24.3	5.9	55.9	13.9
Mean ± SEM	452.7 ± 31.6	89.5 ± 2.0	5.0 ± 1.0	18.8 ± 1.4	7.58 ± 1.8	51.8 ± 1.7	21.7 ± 2.0

#### 2.5.3 Spindle scoring and selection

Sleep spindle events were visually identified as a train of 11–16-Hz waves longer than 500-ms, per the definition of Gibbs and Gibbs (Gibbs, 1950). Fast spindles (>13 Hz) that exhibited a symmetric bilateral distribution over at least three electrodes of centro-parietal areas were selected. In some cases, sleep spindles are associated with Slow Wave Activity (SWA), especially in the first half of the sleep cycles. To make sure that we investigate networks specifically related to the sleep spindle and not the SWA they associate with, all spindles here were 1) selected from NREM2 that by definition is not associated with SWA and moreover 2) were preceded and followed by at least 1 s of relatively silent EEG background i.e. not preceded or followed by artefacts or other electrophysiological events e.g. KCs, vertex and delta waves. For this reason, the number of annotated events for each subject (Table 1) does not reflect spindle density.

The annotation of events was performed manually by three independent raters (DFS, MK and GKK) and observed differences were resolved by a consensus. Event channels were created by manual cursor marking (Neuroscan, Charlotte, NC, USA) and applied to all EEG channels. Moreover, the PZ electrode records were used as reference for marking the sleep spindles’ maximal amplitude peaks and used for the calculation of peak-frequency in the event-related time-frequency analysis. Noisy EEG periods were excluded from the analysis whereas only artefact free sleep spindle epochs were selected.

#### 2.5.4 Event-related time-frequency analysis

Fine-grained Time Frequency Analysis (TFA) was calculated using a custom-made MATLAB-based tool (The Mathworks, Natick, MA, USA) developed at the Neurophysiology Unit, University of Patras, Greece. TFA analysis was estimated using event-related FFT-based transforms centred (t = 0 s) on the annotation marker for a time window of [-1, 2] sec, using a window of 2048 samples with an overlap of 2032 samples and a frequency step of 0.03 Hz. For the TFA calculation a Hamming window was used. The power dimension of the TFA is represented in absolute values and displayed in a linear colour scale (Suppl Fig. 1). TFA for all subjects was estimated using the PZ electrodes. The maximum and approximate upper-lower slope in power have been manually and arbitrarily annotated on the spectrograms. These limits are dependent to the resolution of the FFT calculation.

#### 2.5.5 Subject-specific connectivity

EEG-connectivity values for each subject derived using a methodology based on the imaginary part of coherency (Nolte et al., 2004), that we developed for the subject-specific study of graphoelements (Fig. 1), described in a recent study (Sakellariou et al., 2016). In the latter, interregional interaction levels over time and frequency derive utilising techniques by Nolte and colleagues (Nolte et al., 2004) while the EEG reference and volume conduction problems are discussed.

A Custom-built MATLAB-based (The Mathworks, Natick, MA, USA) tool was used for the estimation of all connectivity and network analyses. The relevant code and support for its implementation can be provided by the corresponding author upon request.

#### 2.5.6 Thresholds and statistical significance

Subject-specific connectivity values were estimated by bootstrap-based statistical analysis using a α = 0.05 level of significance with an approximation of *Z=*1000 number of bootstrap resampled estimates. The typical value of Z repetitions is between 25 and 100 (Efron and Tibshirani, 1994). Specifically for the frequency data of a pair of electrodes for a *κ* spindle epoch $Si\left(f\right),\;Sj(f)$, random samples ${Si}^{⋆}\left(f\right),{Sj}^{⋆}(f)$ are drawn using a pseudo-random number generator with replacement from $Si\left(f\right)=\{d_{i}\left(f,1\right),\ldots ,d_{i}(f,n)\}$ and $Sj\left(f\right)=\{d_{j}\left(f,1\right),\ldots ,d_{j}(f,n)\}$ of n periodograms over all *K* trials in a 1-by-1 scheme in order to populate the model with *K*-resampled trials. *Z* ${Coh}_{ij}^{⋆}(f)$ bootstrap statistics derive from a *Z* number of repetitions of the above procedure. For the confidence interval estimation of a given *a* level of significance, where $P_{\left({Coh}_{l}({{|Coh}_{ji}(\omega )|}^{2})\leq {{|Coh}_{ji}(\omega )|}^{2}\leq {Coh}_{u}({{|Coh}_{ji}(\omega )|}^{2}\right)}=1-a$, the percentiles of the ordered distribution of all bootstrap estimates are calculated (Zoubir, 2005).

In brain connectivity analysis, threshold values are often arbitrarily determined and networks should ideally be characterised across a broad range of thresholds (Rubinov and Sporns, 2010). The cross-subject network findings are presented for low (0.3), medium (0.4) and high (0.5) threshold cutoff values for the averaged imaginary part of coherency, as derived using the methodology in Section 2.2. These threshold values were chosen arbitrarily to emphasize contrasts of the network.

Being a dimensionality reduction technique, PCA aims to represent the data in only a few dimensions (i.e. PCs), while the discarded information is associated with the weakest and least correlated variables in the data. The latter can often safely be assumed to correspond to noise, if the first few PCs capture about >80% of the overall data variance. For cases of numerous non-zero coefficients, an ad hoc way to reduce the number of used variables in standard PCA is to artificially set coefficients with absolute values smaller than a threshold to zero.

## 3 Results

As proof of concept we have applied the described cross-subject methodology to fast sleep spindle events acquired from 10 healthy young adult subjects (Table 2). The subject-specific connectivity data for subjects 01–05 were presented in Sakellariou et al., 2016. In this study, connectivity data for subjects 05–10 were calculated. Moreover, the subject-specific EEG networks for all 10 subjects were estimated to specifically correspond to the fast sleep spindle peak frequency of each subject, as described in the methods (Suppl. Fig. 1, Table 1).

### 3.1 PCA and IPM-SPCA performance

For the PCA and IPM-SPCA calculation, the eigenvectors of the covariance of

$G\in R^{n\times m}$ were estimated using Singular Value Decomposition (Strang et al., 1993), where *n* rows correspond to subjects and *m* columns to the connectivity values of each pair of electrodes after subject-specific mean normalisation. For the IPM-SPCA calculation, the minimum number of non-zero coefficients was adapted according to a threshold of capturing at least 85% of the total variance with three components.

The first three PCs of standard PCA (i.e. the positive contributions of variables with eigenvalues larger than 1), captured 66.4% of the overall data variance. The first principal component (PC1) accounted for 29.3%, PC2 for 20.6% and PC3 for 16.9% of the overall variance (Fig. 2, top left). In the boxplot representation of component scores, three outliers are evident for PC2, while non-symmetrical distribution of scores around the mean was observed for all PCs (Fig. 2, bottom left). Due to the above reasons the representation of the data through standard PCA projections is not considered satisfactory.

The first three Sparse Principal Components (sPC) of IPM-SPCA accounted for 87% of the data variance. Specifically, sPC1, sPC2 and sPC3 accounted for 62.5%, 11.7% and 12.7% of the overall variance respectively, outperforming standard PCA (Fig. 2, top right). The boxplot representation of IPM-SPCA showed symmetrical distribution of scores around the mean for sPC1 and sPC2 and no outliers in any of the SPCs (Fig. 2, bottom right). Yet, SPCA approaches constitute no-closed form expressions (Zou et al., 2006). Therefore, it is essential to compare findings between the standard PCA and IPM-SPCA approaches and validate these findings through a classical framework, that here is the cross-subject network.

### 3.2 Cross-subject network and network modules

**Fig. 3:**
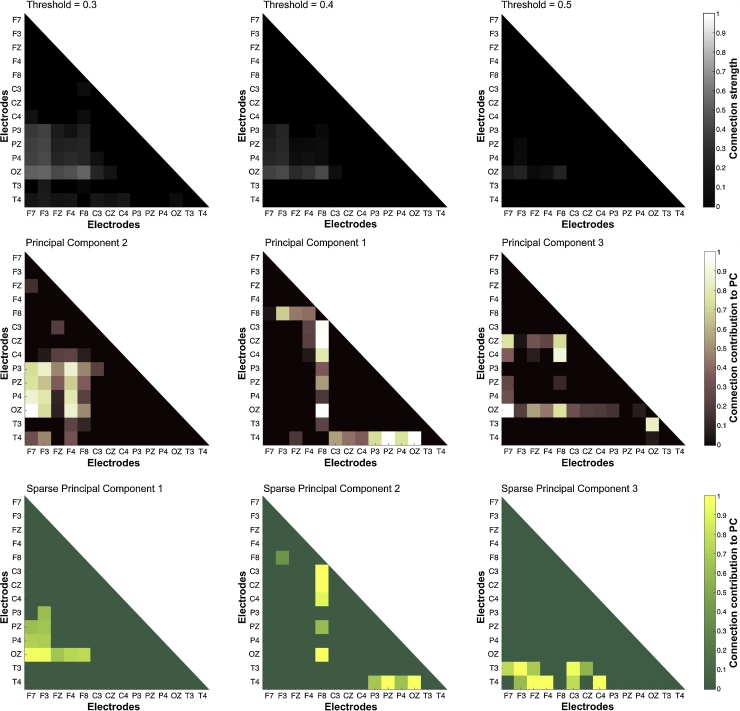
Cross-network, PCA and IPM-SPCA contributions. (Top) Topographies of the cross-network for three cutoff threshold values. The global EEG reach of the sleep spindle is evident at low cutoff values (top left) a strong network module is revealed with medium thresholding and involves occipito-parietal (OZ, P3, PZ, P4) and left-frontal areas (F7, F3) (top - middle). For high threshold values, OZ appears to be the most strongly connected node of the network (top - right). (Middle) Heatmap representation for the contributions of variables on the three larger PCs of standard PCA. PC1 (29.3% of variance) indicates a significant role of T4 and F8 for the fast sleep spindle network, while PC2 and PC3 (20.2% and 16.9% of the variance) are in line with cross-network findings. (Bottom) Contributions of variables on the IPM-SPCA components. The non-zero coefficients of IPM-SPCA confirm the above finding by capturing 87% of the variance and highlight their most important features: a prominent occipito-parietal with left-frontal network module (sPC1 62.5% of the variance), and the importance of OZ and T4 nodes (sPC2 11.7% of the variance) for the EEG spindle network. The importance of T4 is further highlighted by sPC3 (12.4% of the variance).

The cross-subject sleep spindle network is presented for low, medium and high cut-off thresholds according to methods (Fig. 3- top). A diffuse network pattern is evident for low threshold values (Fig. 3 – top left), whereas an interaction pattern between the OZ, P3, PZ, P4 and the F3, F7 areas is evident with medium cut-off values (Fig. 3 – top middle). With high thresholding, this prominent interrelation pattern is further highlighted (Fig. 3 – top center and right).

PCA modules highlight the interconnectedness of F8 and T4 nodes with the rest of the network (PC1), the strong interrelation between OZ, P3, PZ, P4 and F3, FZ, F4 (PC2) and the prominent interconnectedness of OZ node (PC3) (Fig. 3 – middle row). However, many of the PCs’ coefficients take non-zero values e.g. F8 appears interconnected with almost all the sensors in PC1 (Fig. 3 – middle row, center). This makes interpretation of results difficult and outline the need for a SPCA approach.

For the IPM-SPCA, sPC1 strongly suggests the existence of a network module interrelating OZ, P3, PZ, P4 with the F7, F3, F4, F8 nodes by accounting for 62.5% of the overall variance. Furthermore, sPC2 and sPC3 highlight the importance of F8, T4 nodes as branching points of the network (Fig. 3 - bottom). IPM-SPCA network modules are mostly consistent with the PCA findings and the classical framework provided by the cross-subject network.

### 3.3 Node influence metrics

**Fig. 4:**
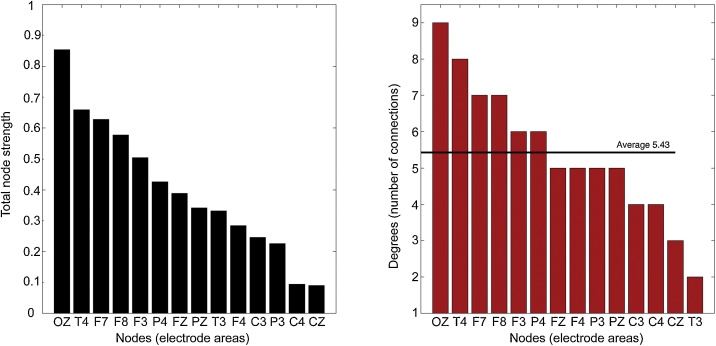
Node strengths and degrees. The degrees of nodes are generally correlated with the NS findings showing OZ, T4 and F7 to hold the most numerous connections.

**Fig. 5:**
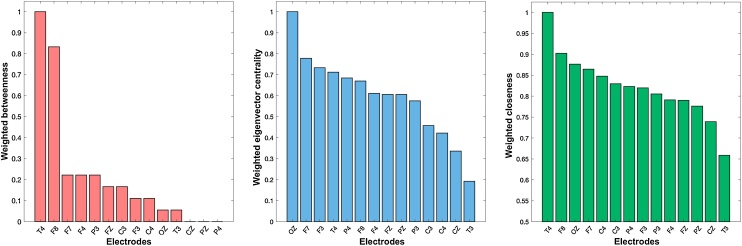
Node centrality measures. Weighted betweenness (left) reveals the T4-F8 area to be an important inter-connector for the spindle network, while the weighted eigenvector centrality (center) suggests the OZ and F3-F7 areas to be the most influential nodes.

Further insight to the structure of the cross-subject network was provided by the node analysis. As far as global networks patterns are concerned and for the 0.5 cut-off value, the OZ node appeared to be the most connected node of the network, both in terms of node strengths and degrees (Fig. 4). T4, F7 and F8 appeared to also be strongly interconnected nodes. From a network theory standpoint, to investigate further the functional role of the above nodes in the network we calculated the weighted closeness, betweenness and eigenvector centralities. Specifically, the T4 and F8 appear to have very high betweenness and closeness values compared to the rest of the areas, suggesting that the right front-temporal areas play a crucial role as intermediate connectors for the rest of spindle network nodes (Fig. 5- left). Moreover, OZ, F7 and F3 were characterized as prestigious nodes in graph-theoretical terms, by taking the highest eigenvector cntrality values (Fig. 5 - center). It is of note that, those areas where not found important as intermediate connectors in the betweenness analysis showing that their authority is transmitted through intermediates, that possibly are the T4-F8 nodes as showed above. Nonetheless, the above model should be viewed as an approximation since it does not involve any informed assumptions about the involvement of deeper structures in the spindle network while all electrical activities are captured at the surface EEG sensor space.

### 3.4 Fast sleep spindle neurophysiological findings

**Fig. 6:**
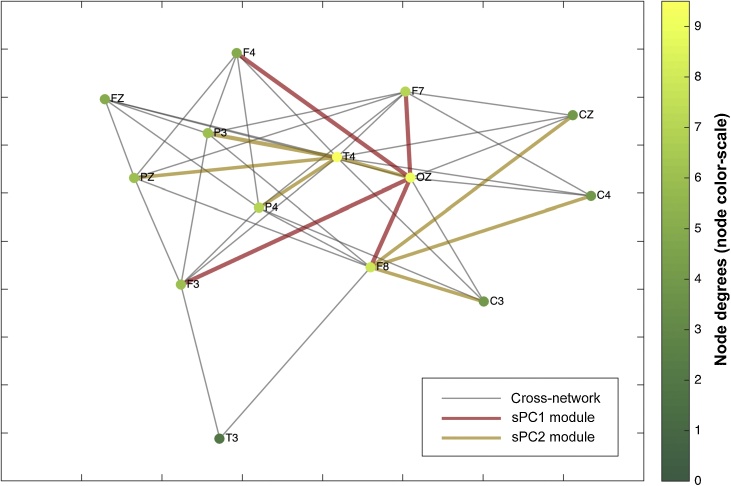
Cross-network and IPM-SPCA graph. The connections of the spindle network are depicted in grey edges (Fig. 3 – top left) and derived from the cross-subject network at low threshold. The two most important network modules account for ∼75% of the cross-subject variance and are depicted in red and brown edges, per sPC1 and sPC2 (Fig. 3 – bottom left and middle). The degrees (i.e. number of connections of nodes) of each area are expressed in the graph on the right side of each electrode’s name in yellow and green colours. The positioning of the nodes was determined according to force-directed placement for undirected graphs, in order to reflect inherent symmetry and centrality features of the system (Fruchterman and Reingold, 1991). OZ, T4 and F8 are the nodes with the highest degrees (nodes in yellow).

The fast sleep spindle network investigation in 10 healthy subjects revealed pilot findings on the prominent involvement and centrality features of occipital-posterior midline areas, along with a possibly important network module that corresponds to occipito-parietal and frontal areas (Fig. 6- in red). Moreover, the right frontal-temporal areas appear to act as branching points in the fast sleep spindle network (Fig. 6 – in brown), which is also evident in the betweenness analysis. Nevertheless, these results serve here as proof of concept and should be considered preliminary as far as the functional EEG networks of the sleep spindle are concerned.

## 4 Discussion and conclusions

In this study, we suggest a strategy for the network investigation of EEG graphoelements across subjects. PCA and IPM-SPCA, managed to capture 66.4% and 87% of the overall variance, in the fast sleep spindles from 10 subjects. The performance of the IPM-SPCA was satisfactory from a methodological point of view.

Pilot neurophysiological findings indicated a module of occipito-parietal and frontal regional interrelation. More specifically, the prominence of OZ and F7-F3 areas was revealed in the fast sleep spindle network. These areas may interact directly during the spindle occurrence however the involvement of deeper structures in this network cannot be ruled out, since sleep spindles are generated through cortico-thalamic oscillations while it has been shown that during NREM sleep cortico-cortical connectivity is much reduced (Massimini et al. Science 2005). Moreover, the right front-temporal areas (F8-T4) appear to act as important regulators for the distribution of information from the prominent nodes to the rest of the network.

Various topographic techniques, including frequency and phase spectra, topographic maps, relative wavelet entropy and covariance are potentially important brain state-dependent measures of neocortical dynamics (Frantzidis et al., 2014; Murias et al., 2007; Srinivasan, 1999; Thatcher et al., 1987). Moreover, phase synchronization and consequently EEG coherency estimates (Mezeiová and Paluš, 2012), can potentially elucidate the formation of dynamic links in the large-scale integration of brain function (Varela et al., 2001).

The sleep spindle has long been considered a global event (Achermann and Borbély, 1998; Andrade et al., 2011; Schabus et al., 2007). Recent studies with intracranial depth EEG however, suggest that spindles are rather local events. Fast sleep spindles were found to be restricted to centro-parietal areas in 13 epilepsy patients (Andrillon et al., 2011). In a similar study of 8 epilepsy patients, sleep spindles with local characteristics were detected in all cortical regions including the temporal and occipital cortex suggesting that their co-occurrence and propagation patterns probably follow a complex brain-wide spatial organization level (Piantoni et al., 2016). Any comparisons with the latter findings are difficult to draw, not only because the placement of iEEG electrodes differed across patients but also because simultaneous scalp EEG recordings were not performed. In a recent study of 35 epilepsy patients that included simultaneous EEG and iEEG, scalp spindles were found to be accompanied by widespread cortical increases in sigma band energy (10–16 Hz) with the highest percentages being observed in the frontal-parietal lateral and mesial cortex (Frauscher et al., 2015). The latter are in line with the network module findings here. However, we cannot validate the prominent involvement of OZ showed here since there was no coverage for occipital areas in the above study.

This study was limited to fast sleep spindles from the second stage of non-REM sleep. Fast and slow spindles have been found to differ in various respects, such as their circadian and homeostatic regulations, pharmacological reactivity, development in infancy, evolution during ageing, modulation during menstrual cycle and pregnancy and intriguingly, their association with general cognitive capabilities and memory processing, to name a few (De Gennaro and Ferrara, 2003; Watson and Buzsaki, 2015). It is therefore questionable whether slow and fast spindles reﬂect the activity of the same generator and network (De Gennaro and Ferrara, 2003). Furthermore, spindles from light and deeper sleep appear to have substantially different neurophysiological characteristics and functions (Andrillon et al., 2011). SWS is associated with reduction in spindle occurrence and spindle spectral frequency possibly due to the stronger underlying thalamocortical hyperpolarization. However, an evolution from posterior-fast to anterior-slow generators has been suggested to occur during spindles (Dehghani et al., 2011). Additionally, while processing of external information in light sleep can occur, it is suppressed in SWS. Spoormaker et al. (Spoormaker et al., 2011) proposed that upon sleep onset, the large-scale functional brain network progresses through light sleep towards a globally distributed network, optimal for the global transfer of information. This network finally segregates in network modules at SWS to reprocess information in an isolated manner. Such findings suggest that the networks associated with spindles in light and deep non-REM sleep are not necessarily identical. Also, sleep spindles in the first half of the night are closely associated with slow wave activity (SWA). This functional difference across sleep cycles is also reflected in the neuroendocrine activity which also differs between early and late nocturnal sleep. For these reasons, we particularly examined fast sleep spindles from the second stage of non-REM sleep that by definition SWA is not part of it.

Although the selection criteria for sleep spindles as mentioned above are sufficiently consistent in the micro-state level, the homogeneity of the investigated events can be considered limited as far as sleep cycles are concerned. Specifically, sleep spindles in silent background are more likely to occur in the second half of the night sleep as the SWA decreases in later sleep cycles. This functional difference across cycles is also reflected in neuroendocrine activity that also differs between early and late nocturnal sleep. Nonetheless, the pilot results here are representative of the network patterns as seen across sleep spindles from all sleep cycles of NREM2, since we only chose trials from specifically that stage where SWA is not part of it.

With regard to methods, here we made use of PCA, a dimensionality reduction techniques that assumes approximate normality of the input space distribution (Blanchard et al., 2007). In many cases however, data do not correlate linearly and thus relevant PCA findings may be a poor interpretation of the natural variance of a system. Nonetheless, here we compared standard PCA findings with an IPM-SPCA model to validate findings and the percentage of captured data variance constitutes the relevant down-projections as a sufficient representation of possibly important spatial features. Kernel PCA (Schölkopf et al., 1997) may also be an interesting approach as far as the above limitations are concerned, by allowing for mapping the data to a high-dimensional feature space. From a neurophysiological point of view, findings here can only be considered preliminary due to the experimental nature of this methodology and importantly the number of subjects. With that in mind, the network module that involves lateral occipito-parietal and frontal areas may possibly be part of the global fields into which local networks may be embedded into (Nunez et al., 2015). The prominence of OZ areas as seen here, is generally not aligned with the literature on spindles. However it is important to note that, the node findings of this study do not relate to measures of source or maximal activation, but rather to estimates of network influence. Being amplitude-invariant and phase-dependent, this approach is aimed to provide with a view supplementary to the established EEG-topography methods, and therefore relevant findings should be carefully compared to other techniques.

In EEG studies, the common connectivity patterns across a group of subjects are often estimated using the grand average. Here, we proposed a novel methodology for the extraction of connectivity representations of a group with an emphasis on cross-subject variance. This method provides a complementary platform to explore and visualize EEG connectivity results, and provide with further insight for important EEG questions e.g. differences in the functional networks of fast and slow as well as S2 and SWS sleep spindles, as suspected in previous studies (Spoormaker et al., 2011; De Gennaro and Ferrara, 2003). Moreover, EEG functional networks and the topography of various EEG graphoelements such as the KC and SWD appear to play a key role in a variety of brain diseases (De Gennaro et al., 2017; Nunez et al., 2015; Wamsley et al., 2012; Chang et al., 2009; Yoshinaga et al., 1996; Thatcher et al., 1986; Gotman, 1981). Exploration of relevant large scale datasets using the proposed tools may be able to reveal robust network biomarkers to assist diagnosis in neurological conditions.

## Funding

DFS is funded by the Medical Research Council Confidence in Concept grant (#MC_PC_16048). GKK was funded by ARMOR EU (FP7/2007-2013) agreement number 287720. MR is funded by Medical Research Council (Programme grant MR/K013998/1), Engineering and Physical Sciences Research Council (Centre for Predictive Modelling in Healthcare EP/N014391/1) and by the NIHR Biomedical Research Centre for Mental Health at the South London and Maudsley NHS Foundation Trust.

## Authors and Contributors

GKK and DFS conceived the project. DFS designed, developed and applied all methodologies. Sean Higgins performed the sleep staging analysis. DFS wrote the manuscript. Data was collected by Maria Tsirouda and DFS, and Andrew Koupparis and Vasileios Kokkinos. GKK, MK and MR amended and enriched the text. GKK, MK and MR supervised the study and reviewed the manuscript.

## Competing financial interests

The authors declare no competing financial interests.

## Figures and Tables

**Figure:**
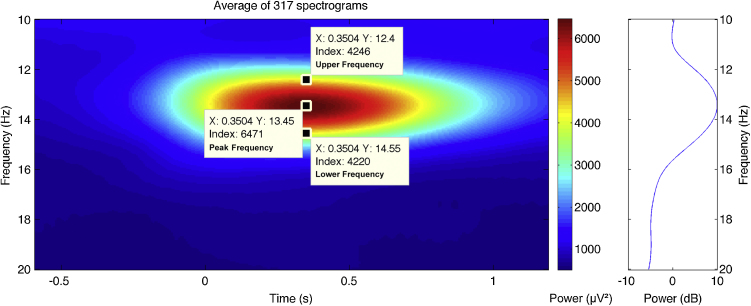


**Figure:**
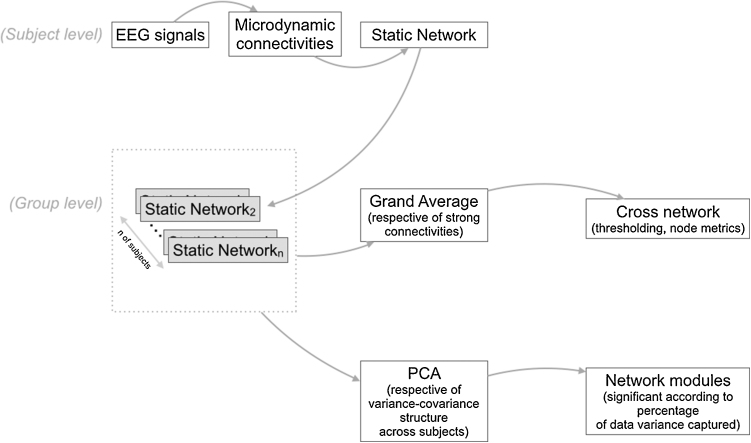


## References

[bib0005] Achermann P., Borbély A.A. (1998). Coherence analysis of the human sleep electroencephalogram. Neuroscience.

[bib0010] Andrade K.C.C., Spoormaker V.I., Dresler M., Wehrle R., Holsboer F., Sämann P.G., Czisch M. (2011). Sleep spindles and hippocampal functional connectivity in human NREM sleep. J. Neurosci..

[bib0015] Andrillon T., Nir Y., Staba R.J., Ferrarelli F., Cirelli C., Tononi G., Fried I. (2011). Sleep spindles in humans: insights from intracranial EEG and unit recordings. J. Neurosci..

[bib0020] Ayoub A., Mölle M., Preissl H., Born J. (2012). Grouping of MEG gamma oscillations by EEG sleep spindles. NeuroImage.

[bib0025] Beniczky S., Hirsch L.J., Kaplan P.W., Pressler R., Bauer G., Aurlien H., Brøgger J.C., Trinka E. (2013). Unified EEG terminology and criteria for nonconvulsive status epilepticus. Epilepsia.

[bib0030] Berry R.B., Budhiraja R., Gottlieb D.J., Gozal D., Iber C., Kapur V.K., Marcus C.L., Mehra R., Parthasarathy S., Quan S.F., Redline S., Strohl K.P., Davidson Ward S.L., Tangredi M.M. (2012). American Academy of Sleep, M., 2012. Rules for scoring respiratory events in sleep: update of the 2007 AASM Manual for the Scoring of Sleep and Associated Events. Deliberations of the Sleep Apnea Definitions Task Force of the American Academy of Sleep Medicine. J. Clin. Sleep Med..

[bib0035] Blanchard G., Bousquet O., Zwald L. (2007). Statistical properties of kernel principal component analysis. Mach. Learn..

[bib0040] Cadima J., Jolliffe I.T. (1995). Loading and correlations in the interpretation of principle compenents. J. Appl. Stat..

[bib0045] Chang E.F., Nagarajan S.S., Mantle M., Barbaro N.M., Kirsch H.E. (2009). Magnetic source imaging for the surgical evaluation of electroencephalography-confirmed secondary bilateral synchrony in intractable epilepsy. J. Neurosurg..

[bib0050] Colrain I.M. (2005). The K-complex: a 7-decade history. Sleep.

[bib0055] Contreras D., Destexhe A., Sejnowski T.J., Steriade M. (1997). Spatiotemporal patterns of spindle oscillations in cortex and thalamus. J. Neurosci..

[bib0060] Crowley K., Trinder J., Kim Y., Carrington M., Colrain I.M. (2002). The effects of normal aging on sleep spindle and K-complex production. Clin. Neurophysiol..

[bib0065] De Gennaro L., Ferrara M. (2003). Sleep spindles: an overview. Sleep Med. Rev..

[bib0070] De Gennaro L., Gorgoni M., Reda F., Lauri G., Truglia I., Cordone S., Scarpelli S., Mangiaruga A., D’Atri A., Lacidogna G., Ferrara M., Marra C., Rossini P.M. (2017). The fall of sleep K-Complex in Alzheimer disease. Sci. Rep..

[bib0075] Dehghani N., Cash S.S., Halgren E. (2011). Topographical frequency dynamics within EEG and MEG sleep spindles. Clin. Neurophysiol..

[bib0080] Dijk D.J., Hayes B., Czeisler C.A. (1993). Dynamics of electroencephalographic sleep spindles and slow wave activity in men: effect of sleep deprivation. Brain Res..

[bib0085] Dijkstra E.W. (1959). A note on two problems in connexion with graphs. Numer. Math..

[bib0090] Efron B., Tibshirani R.J. (1994). An Introduction to the Bootstrap.

[bib0095] Fogel S.M., Smith C.T. (2011). The function of the sleep spindle: a physiological index of intelligence and a mechanism for sleep-dependent memory consolidation. Neurosci. Biobehav. Rev..

[bib0100] Frantzidis C.A. (2014). Functional disorganization of small-world brain networks in mild Alzheimer’s Disease and amnestic Mild Cognitive Impairment: an EEG study using Relative Wavelet Entropy (RWE). Front. Aging Neurosci..

[bib0105] Frauscher B., von Ellenrieder N., Dubeau F., Gotman J. (2015). Scalp spindles are associated with widespread intracranial activity with unexpectedly low synchrony. NeuroImage.

[bib0110] Freeman (1977). A set of measures of centrality based on betweenness. Sociometry.

[bib0115] Fruchterman T.M.J., Reingold E.M. (1991). Graph drawing by force directed placement. Softw. Pract. Exp..

[bib0120] Gibbs F. (1950).

[bib0125] Gotman J. (1981). Interhemispheric relations during bilateral spike-and-wave activity. Epilepsia.

[bib0130] Halász P., Bódizs R. (2013). Dynamic NREM Sleep Regulation Models. Dynamic Structure of NREM Sleep.

[bib0135] Hein M., Bühler T. (2010). An inverse power method for nonlinear eigenproblems with applications in 1-spectral clustering and sparse PCA. Adv. Neural Inf. Process. Syst..

[bib0140] Joliffe I.T., Morgan B.J.T. (1992). Principal component analysis and exploratory factor analysis. Stat. Methods Med. Res..

[bib0145] Keller C.J., Honey C.J., Mégevand P., Entz L., Ulbert I., Mehta A.D. (2014). Mapping human brain networks with cortico-cortical evoked potentials. Philos. Trans. R. Soc. Lond., B, Biol. Sci..

[bib0150] Knoblauch V., Martens W.L., Wirz-Justice A., Cajochen C. (2003). Human sleep spindle characteristics after sleep deprivation. Clin. Neurophysiol..

[bib0155] Kokkinos V., Kostopoulos G.K. (2011). Human non-rapid eye movement stage II sleep spindles are blocked upon spontaneous K-complex coincidence and resume as higher frequency spindles afterwards. J. Sleep Res..

[bib0160] Kokkinos V., Koupparis A.M., Kostopoulos G.K. (2013). An intra-K-complex oscillation with independent and labile frequency and topography in NREM sleep. Front. Hum. Neurosci..

[bib0165] Kokkinos V., Koutroumanidis M., Tsatsou K., Koupparis A., Tsiptsios D., Panayiotopoulos C.P. (2010). Multifocal spatiotemporal distribution of interictal spikes in Panayiotopoulos syndrome. Clin. Neurophysiol..

[bib0170] Kostopoulos G., Gloor P., Pellegrini A., Gotman J. (1981). A study of the transition from spindles to spike and wave discharge in feline generalized penicillin epilepsy: microphysiological features. Exp. Neurol..

[bib0175] Kostopoulos G.K. (2000). Spike-and-wave discharges of absence seizures as a transformation of sleep spindles: the continuing development of a hypothesis. Clin. Neurophysiol..

[bib0180] Loomis A.L., Harvey E.N., Hobart G.A. (1937). Cerebral states during sleep, as studied by human brain potentials. J. Exp. Psychol..

[bib0185] Meyer C. (2000). Matrix Analysis and Applied Linear Algebra.

[bib0190] Mezeiová K., Paluš M. (2012). Comparison of coherence and phase synchronization of the human sleep electroencephalogram. Clin. Neurophysiol..

[bib0195] Murias M., Webb S.J., Greenson J., Dawson G. (2007). Resting state cortical connectivity reflected in EEG coherence in individuals with autism. Biol. Psychiatry.

[bib0200] Nolte G., Bai O., Wheaton L., Mari Z., Vorbach S., Hallett M. (2004). Identifying true brain interaction from EEG data using the imaginary part of coherency. Clin. Neurophysiol..

[bib0205] Nunez P.L. (1997). EEG coherency. I: statistics, reference electrode, volume conduction, Laplacians, cortical imaging, and interpretation at multiple scales. Electroencephalogr. Clin. Neurophysiol..

[bib0210] Nunez P.L., Srinivasan R., Fields R.D. (2015). EEG functional connectivity, axon delays and white matter disease. Clin. Neurophysiol..

[bib0215] Nunez P.L., Srinivasan R., Westdorp A.F., Wijesinghe R.S., Tucker D.M., Silberstein R.B., Cadusch P.J. (1997). EEG coherency. I: statistics, reference electrode, volume conduction, Laplacians, cortical imaging, and interpretation at multiple scales. Electroencephalogr. Clin. Neurophysiol..

[bib0220] O’Reilly C., Nielsen T. (2014). Assessing EEG sleep spindle propagation. Part 1: theory and proposed methodology. J. Neurosci. Methods.

[bib0225] O’Reilly C., Nielsen T. (2014). Assessing EEG sleep spindle propagation. Part 2: experimental characterization. J. Neurosci. Methods.

[bib0230] Pearson K. (1901). LIII. On lines and planes of closest fit to systems of points in space. Lond. Edinburgh Dublin Philos. Mag. J. Sci..

[bib0235] Piantoni G., Halgren E., Cash S.S. (2016). Spatiotemporal characteristics of sleep spindles depend on cortical location. NeuroImage.

[bib0240] Pizzo F., Ferrari-Marinho T., Amiri M., Frauscher B., Dubeau F., Gotman J. (2016). When spikes are symmetric, ripples are not: bilateral spike and wave above 80 Hz in focal and generalized epilepsy. Clin. Neurophysiol..

[bib0245] Rodenbeck A., Binder R., Geisler P. (2006). A Review of Sleep EEG Patterns. Part I: a Compilation of Amended Rules for Their Visual Recognition According to Rechtschaffen and Kales.

[bib0250] Roland P.E., Hilgetag C.C., Deco G. (2014). Cortico-cortical communication dynamics. Front. Syst. Neurosci..

[bib0255] Rubinov M., Sporns O. (2010). Complex network measures of brain connectivity: uses and interpretations. NeuroImage.

[bib0260] Sabidussi G. (1996). The centrality index of a graph. Psychometrika.

[bib0265] Sakellariou D., Koupparis A.M., Kokkinos V., Koutroumanidis M., Kostopoulos G.K. (2016). Connectivity measures in EEG microstructural sleep elements. Front. Neuroinform..

[bib0270] Schabus M., Dang-Vu T.T., Albouy G., Balteau E., Boly M., Carrier J., Darsaud A., Degueldre C., Desseilles M., Gais S., Phillips C., Rauchs G., Schnakers C., Sterpenich V., Vandewalle G., Luxen A., Maquet P. (2007). Hemodynamic cerebral correlates of sleep spindles during human non-rapid eye movement sleep. Proc. Natl. Acad. Sci. U. S. A..

[bib0275] Schölkopf B., Smola A., Müller K.-R. (1997). Kernel Principal Component Analysis. Kernel Principal Component Analysis.

[bib0280] Sitnikova E. (2010). Thalamo-cortical mechanisms of sleep spindles and spike-wave discharges in rat model of absence epilepsy (a review). Epilepsy Res..

[bib0285] Spoormaker V.I., Czisch M., Maquet P., Jäncke L. (2011). Large-scale functional brain networks in human non-rapid eye movement sleep: insights from combined electroencephalographic/functional magnetic resonance imaging studies. Philosophical transactions. Ser. A Math. Phys. Eng. Sci..

[bib0290] Srinivasan R. (1999). Spatial structure of the human alpha rhythm: global correlation in adults and local correlation in children. Clin. Neurophysiol..

[bib0295] Steriade M. (2006). Grouping of brain rhythms in corticothalamic systems. Neuroscience.

[bib0300] Strang G., Strang G., Strang G., Strang G. (1993). Introduction to Linear Algebra.

[bib0305] Thatcher R.W., Krause P.J., Hrybyk M. (1986). Cortico-cortical associations and EEG coherence: a two-compartmental model. Electroencephalography Clin..

[bib0310] Thatcher R.W., Walker R.A., Giudice S. (1987). Human cerebral hemispheres develop at different rates and ages. Science.

[bib0315] Varela F., Lachaux J.P., Rodriguez E. (2001). The brainweb: phase synchronization and large-scale integration. Nat. Rev..

[bib0320] Vyazovskiy V.V., Achermann P., Borbély A.A., Tobler I. (2004). The dynamics of spindles and EEG slow-wave activity in NREM sleep in mice. Arch. Ital. Biol..

[bib0325] Wamsley E.J., Tucker M.A., Shinn A.K., Ono K.E., McKinley S.K., Ely A.V., Goff D.C., Stickgold R., Manoach D.S. (2012). Reduced sleep spindles and spindle coherence in schizophrenia: mechanisms of impaired memory consolidation?. Biol. Psychiatry.

[bib0330] Wang H., Hernandez J., Mieghem P. (2008). Betweenness centrality in a weighted network. Phys. Rev..

[bib0335] Watson B.O., Buzsaki G. (2015). Sleep, memory & brain rhythms. Daedalus.

[bib0340] Weigenand A., Mölle M., Werner F., Martinetz T., Marshall L. (2016). Timing matters: open-loop stimulation does not improve overnight consolidation of word pairs in humans. Eur. J. Neurosci..

[bib0345] Yoshinaga H., Kobayashi K., Sato M., Oka E., Ohtahara S. (1996). Investigation of bilateral synchronous spike-wave discharge by EEG topography. Brain Topogr..

[bib0350] Zou H., Hastie T., Tibshirani R. (2006). Sparse principal component analysis. J. Comput. Graph. Stat..

[bib0355] Zoubir A.M. (2005).

