# Supplementary material for: Cross-subject network investigation of the EEG microstructure: A sleep spindles study
Source: J Neurosci Methods. 2019 Jan 15;312:16–26. doi: 10.1016/j.jneumeth.2018.11.001 (PMC6327148; doi:10.1016/j.jneumeth.2018.11.001)
Supplement: Supplementary file 1 [file mmc1.docx]

**Suppl Fig 1 | Fast Sleep Spindle Spectrogram** Average spectrogram of fast sleep spindles for subject 6. The maximum and approximate upper-lower power slopes have been manually annotated. These frequency limits, were used for the calculation of spindle-specific networks of each subject. Time-average of all annotated fast sleep spindles (Right) Fast Fourier Transform (FFT) power spectral density distribution.

**Suppl Fig 2 | Pipeline of analysis from subject to group level**
